# Immune profiling of T cells: A comparative analysis of single-cell TCR sequencing using 10X Genomics and Parse Biosciences platforms

**DOI:** 10.1016/j.bbrep.2026.102592

**Published:** 2026-04-19

**Authors:** Nanna Kristjánsdóttir, Kasper Thorsen, Nicolai J. Birkbak, Lars Dyrskjøt

**Affiliations:** a Department of Molecular Medicine, Aarhus University Hospital, Aarhus N, 8200, Denmark; b Department of Clinical Medicine, Aarhus University, Aarhus, 8000, Denmark

## Abstract

Single-cell RNA sequencing (scRNA-seq) is a powerful tool for characterising the immune landscape. Multiple technologies are available, each with distinct specificities and inherent biases. Here, we focus on two technologies—10X Genomics and Parse Biosciences—for the paired profiling of TCR and whole-transcriptome in T cells using matched patient samples. Both platforms generated high-quality data and captured comparable TCR clonal landscapes, with strong concordance for dominant clones. More genes were captured with Parse, including a broader range of non-coding genes and pseudogenes. Although average gene expression was highly correlated across the platforms, feature selection, which retains only the most informative genes, yielded different sets of selected genes. Additionally, many T-cell subset markers varied substantially between platforms. Genes enriched in Parse were typically longer and associated with naive/central-memory T-cell genes, whereas those enriched in 10X were shorter and often associated with cytotoxic/effector genes. These patterns are consistent with differences in capture chemistry. These findings highlight meaningful considerations for platform selection, which should be aligned with study objectives. Parse offers an advantage for studies prioritising a wider range of genes, while 10X may be the apparent choice for detailed characterisation of effector function.

## Highlights

• Comparative analysis of scRNA-seq from matched T cells with 10X Genomics and Parse.
• Both platforms reveal concordant TCR clonal landscapes.
• Parse captures more non-coding genes and pseudogenes.
• 10X enriches for shorter, effector-linked T cell genes.
• Platform choice should align with study goals and biases.

## Graphical abstract

**Figure:**
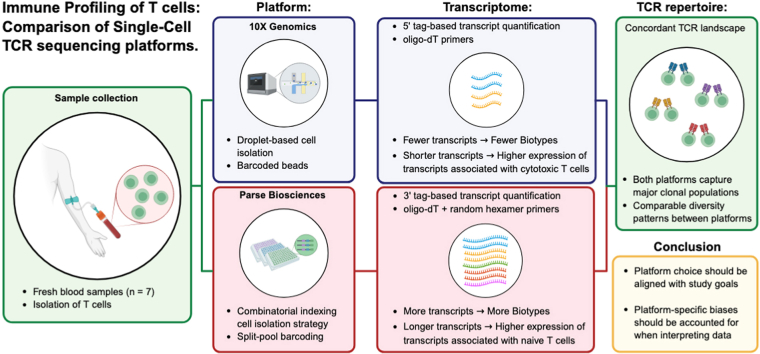

## 1 Introduction

The immune system comprises diverse cell types, each playing distinct roles in defending the body against infections and diseases. Historically, researchers have relied on protein-based techniques to characterise the immune landscape. Methods such as western blotting, immunohistochemistry, flow cytometry, and ELISA laid the foundation for understanding tumour immunology [1]. More recently, sequencing technologies, particularly single-cell RNA sequencing (scRNA-seq), have expanded this toolkit by enabling genome-wide analysis of gene expression. Once considered a niche technique, scRNA-seq is now a standard tool in immunology and disease research, providing high-resolution characterisation of cellular diversity, gene expression, and immune mechanisms [2]. However, as the field advances with new technologies and evolving methods, comparing results across studies becomes increasingly challenging.

Current scRNA-seq technologies vary in cell isolation and barcoding strategies. The most used approaches are microfluidic systems that encapsulate individual cells in droplets with barcoded beads [[3], [4], [5], [6]] or small reaction chambers [7]. Other methods are well-based, where cells are physically isolated into individual wells [[8], [9], [10], [11], [12], [13], [14]] or sequentially labelled using combinatorial barcoding [15,16]. Transcript quantification strategies also differ substantially between methods, some based on full-length sequencing, where reads uniformly span the entire transcript, others using tag-based sequencing, which focuses on capturing either the 5′ or 3’ terminus (Table 1).

**Table 1:** Overview over scRNAseq platforms.

Technology	Cell Isolation Strategy	Barcoding strategy	Transcript Quantification
**CEL-Seq2[**9]	Plate-based (FACS into wells)	Unique molecular identifiers	Tag-based (3′)
**Chromium[**4]	Microfluidics (droplet-based)	Barcoded Beads	Tag-based (3′ or 5′)
**Drop-seq[**5]	Microfluidics (droplet-based)	Barcoded Beads	Tag-based (3′)
**Fluidigm C1[**7]	Microfluidic chambers	Chamber-specific barcoding	Full-length
**inDROP[**6]	Microfluidics (droplet-based)	Barcoded Beads	Tag-based (3′)
**MARS-seq[**10]	Plate-based	Barcoded primers	Tag-based (3′)
**sci-RNA-seq[**16]	Combinatorial indexing	Split-pool barcoding	Tag-based (3′)
**Seq-Well[**14]	Microwell arrays	Beads into individual wells	Tag-based (3′)
**Smart-seq[**8,[11], [12], [13]]	Plate-based (FACS into wells)	No barcoding	Full-length
**SPliT-seq[**15]	Combinatorial indexing	Split-pool barcoding	Tag-based (3′)

T cells are an essential part of the adaptive immune system, and their defence mechanism is based on recognising foreign antigens through their T cell receptors (TCRs). Traditionally, TCR sequencing has been performed using bulk approaches, which are widely used to characterise immune repertoires in health and disease, but only provide an average measurement of T cell characteristics. A comprehensive analysis of T cells requires simultaneous measurement of the TCR and gene expression from individual T cells, which is only possible using single-cell sequencing. Here, we focus on two diverging technologies for characterising T cells: 10X Genomics and Parse Biosciences, each offering unique advantages for T cell characterisation.

The Chromium system is a microfluidics-based droplet system and is widely used in the field. This approach encapsulates individual cells within droplets containing barcoded beads, where cells are lysed, and their mRNA is captured by the barcoded primers for cell-specific tagging of transcripts. While the Chromium system supports both 3′ and 5′ capture chemistries, here we focus on the 5′ workflow utilising the Universal 5′ Gene Expression technology. This approach uses oligo-dT primers and SMART template switching technology to capture full-length cDNA, including complete TCR regions. Then the droplets are lysed, and the tagged cDNA from all cells is pooled, ready for library preparation and sequencing. Although this method typically recovers only ∼10% of the cell's transcripts, this is generally sufficient for robust cell-type identification. However, the requirement for intact, live cells presents challenges for clinical studies where samples may be fragile and have limited availability [4].

Parse Biosciences offers a newer, instrument-free alternative based on split-pool combinatorial barcoding. The Evercode™ method begins by sample fixation to preserve cell structure and lock the RNA inside the cell, thereby preventing its degradation. This fixation stabilises the cells as reaction vessels, eliminating multiplets and enhancing data integrity. The fixed samples are stable for up to 6 months at −80 °C, providing flexibility and scalability for sample collection and making the method ideal for time-course studies and multiplexing. The process involves assigning unique barcodes to fixed samples through an in-cell reverse transcription reaction in individual wells, using a combination of oligo-dT and random hexamer primers. This approach enables broader gene coverage, not limited to polyadenylated RNAs. Then, the cells are pooled and redistributed into a multi-well plate for additional barcoding rounds with in-cell ligations. This iterative process generates millions of unique barcode combinations, ensuring each cell has a distinct label. The labelled cells are then divided into sublibraries for library preparation and sequencing. A key advantage of this method is the reduction of batch effects and technical variability, as samples are mixed early in the workflow [15].

Previous studies have compared scRNA-seq methods from the two manufacturers [17,18], but using the Chromium 3’ kit, which does not utilise template switching. One study, using peripheral blood mononuclear cells from healthy individuals, found that cell type frequencies were unaffected by method choice, but gene length and GC content were platform-specific [17]. Another study used mouse thymus as a model tissue and concluded that 10X provided less technical variability and more precise annotation of biological states [18]. However, a direct comparison of template-switching and combinatorial barcoding, in combination with TCR sequencing, is lacking.

Here, we present a comparison of the 10X Genomics and Parse Biosciences platforms for characterising circulating T cells. We examine similarities and differences in sample quality, transcriptomic profiling, and T-cell characteristics. To our knowledge, this is the first study to compare the combined immune profiling of T cells using both TCR and whole transcriptome across the two platforms. Such combined profiling enables direct linking of clonal structure to functional cell states, allowing deeper interpretation of T-cell heterogeneity than transcriptomic data alone. By benchmarking two platforms that deliver these capabilities, our study can provide researchers with evidence-based guidance when selecting an appropriate platform for integrated TCR and transcriptomic profiling in T-cell studies.

## 2 Materials and methods

### 2.1 Patients

Samples in this study were obtained from seven patients with bladder cancer (MIBC, n = 5; NMIBC, n = 2). All patients were enrolled in larger research projects and provided written informed consent prior to inclusion. The projects were approved by the National Scientific Ethical Committee (#1706501 and EudraCT number: 2019-001679-36). Clinical information about the patients is not reported as it falls outside the scope of this manuscript. Samples were collected from the Department of Urology at Aarhus University Hospital (Denmark), during scheduled clinical visits. All samples were transferred immediately to the laboratory for processing.

### 2.2 Sample preparation and library production

T cells were isolated from 1 mL of blood using EasySep(TM) Direct Human T Cell Isolation Kit (STEMCELL(TM)) according to protocol. The sample's concentration, viability, and aggregation were measured using Nucleocounter (R) NP-3000(TM). The target concentration of 7-12x10^5 cells/mL was chosen to align with the manufacturer's (10X's [19]) optimal loading recommendation, which maximises recovery while minimising multiplets. Samples below this range were centrifuged for 5 min at 250G at room temperature and resuspended in PBS to a final concentration of 1x10^6 cells/mL to ensure comparable cell input conditions. Volumes corresponding to a target recovery of 5000 cells were directly taken from each sample and put into the Chromium Next GEM Single Cell 5′ Reagent Kits v2 (Dual Index) workflow from 10X Genomics. The 5000-cell target was selected based on prior experience and a previously published study using this protocol [20]. Libraries were made according to protocol. The remaining single-cell suspensions were immediately fixated using Evercode(TM) Cell Fixation v2.1.1 from Parse Biosciences according to protocol [21]. Libraries with a target recovery of 12,500 cells were created using Evercode(TM) TCR, Human TCR + WT v1.0.3 from Parse Biosciences, according to protocol. The quality of all libraries was measured using Tapestation HS-D5000, and the quantity of the final libraries was measured using Qubit. All libraries were paired-end sequenced using NovaSeq 6000 S2 flowcell with 216 bp Read 1 and 90 bp Read 2 according to the manufacturer's instructions (Illumina).

### 2.3 Raw data processing and quality control

The raw reads were trimmed according to the library type and read orientation. For 10X Genomics libraries, Read 1 was trimmed to retain bases 1-26, both wild-type (WT) and TCR libraries. For Parse Biosciences libraries, Read 1 from WT samples was trimmed to retain bases 1-100, while the TCR libraries were left untrimmed. Read 2 was used without trimming for all libraries from both platforms.

The 10X data was pre-processed (demultiplexed, reads aligned and filtered, barcodes and UMIs counted, and TCR clones determined) using Cell Ranger (10X Genomics). The Parse data was pre-processed using split-pipe v1.1.2 with default settings (Parse Biosciences). The data was loaded and filtered individually using Scanpy (v.1.10.3) [22], Pandas (v2.2.3), and NumPy (v1.26.4). First, cells with <100 unique transcripts and transcripts present in fewer than 20 cells per sample were removed using scanpy.pp.filter_cells and scanpy.pp.filter_genes, respectively. Second, quality control (QC) metrics, including total counts, the number of transcripts detected, the percentage of counts in the top 20 expressed transcripts, and mitochondrial content (genes starting with ‘MT-’), were calculated using the scanpy.pp.calculate_qc_metrics function. Third, data outliers were identified using a thresholding approach based on the median absolute deviation (MAD) as described earlier [23]. A MAD threshold of 5 was applied on the following parameters: the logarithm of total counts per cell, the logarithm of the number of genes detected per cell, and the percentage of counts attributed to the top 20 expressed genes. Any cells flagged as outliers in any of these metrics were excluded from downstream analysis, and cells with more than 10% mitochondrial content were also excluded. Lastly, TCR sequences and all relevant information from TCRseq were attached to the data, and cells missing a TRA or TRB sequence were removed.

### 2.4 Transcript expression and harmonisation

Highly variable transcripts were identified using a dispersion-based method. First, the data were normalised to 10,000 total counts per cell using scanpy.pp.normalize_total and log-transformed using scanpy.pp.log1p. Secondly, genes are binned by mean expression, and the dispersion of each gene within that bin is normalised by subtracting the dispersion of that bin and dividing by the standard deviation of that bin using scanpy.pp.highly_variable_genes. Transcripts with a mean higher than 0.0125 and lower than three and a normalised dispersion higher than 0.25 are selected as highly variable.

The top 1000 genes by total counts were found using scanpy.pp.calculate_QC_metrics.

Ensembl [24] gene identifiers were used to harmonise the gene identifications. The 10X data was given Ensembl gene identifiers using PyEnsembl (v2.3.13) and Ensembl Release 98, while Parse already provides these identifiers from Ensembl Release 109 as part of the split-pipe output. The data was filtered to only include genes included in Ensembl Release 98 and 109. The gene biotypes were found using PyEnsembl and the Ensembl Release 109.

Expressions of T cell subtype-related genes were visualised using the scanpy.pl.umap, and summary statistics were calculated using numpy. The transcript length was determined using Ensembl Release 109 and the canonical transcript.

### 2.5 Batch correction

The 10X and Parse data were processed separately using the scVI framework (v1.2.0) [25,26], a probabilistic deep-learning method for integrating single-cell RNA-seq data, with the samples treated as batch variables. The scVI model was initialised with the default parameters: latent dimensionality = 10, hidden units = 128, hidden layers = 1, dropout rate = 0.1, and gene dispersion modelled at the gene level. The model was trained using parameters adapted from the HLCA integration workflow [27] for a maximum of 500 epochs, with validation checks performed after each epoch. The validation evidence lower bound (ELBO) was monitored after each epoch, and the model stopped if the ELBO did not improve for 10 consecutive epochs. A learning rate reduction factor of 0.1 was applied when the learning plateaued.

Batch effects were visualised using UMAP embeddings computed using scanpy.tl.umap and plotted using scanpy.pl.umap. Neighbourhood graphs were constructed with scanpy.pp. neighbours, using 30 principal components obtained using scanpy.tl.pca for the uncorrected data, and the scVI latent representation for the corrected data.

### 2.6 TCR clonal analysis

Clonal overlap between platforms was assessed using the TCR annotations generated during preprocessing. For cells with multiple TRA or TRB, the first TRA and TRB chains listed were used to define the TCR clonotype. Clones were classified as follows: Common - identical paired TRA-TRB sequences detected in both datasets; Partly - either (i) only one chain (TRA or TRB) was shared between datasets, or (ii) both chains were present in both datasets but not observed as the same pair; Unique - clones for which neither TRA or TRB was observed in the other dataset. TCR repertoires were visualised using packcircles (v0.3.6) and ggplot2 (v3.5.1) in R (v4.3.3).

TCR diversity indexes were calculated as follows:

Shannon diversity index: $H=-\sum_{i=1}^{S}p_{i}*\log\left(p_{i}\right)$ where $p_{i}$ is the relative abundance of a clone i, and S is the number of observed clones.

Pielou's evenness index is derived by normalising Shannon's entropy: $J=\frac{H}{\log\left(S\right)}$

Simpson's diversity index: $D=1-\sum_{i=1}^{S}p_{i}^{2}$

Gini index: $G=\frac{2\sum_{i=1}^{S}i*p_{\left(i\right)}}{S}-\frac{S+1}{S}$

Chao1 richness estimator: $Chao1=S_{obs}+\frac{f_{1}^{2}}{2f_{2}}$ where $S_{obs}$ is the number of observed transcripts, $f_{1}$ is the number of singletons, and $f_{2}$ is the number of doubletons.

D50 index: $D50=\frac{n_{50}}{S}*100$ where $n_{50}$ is the minimum number of clones required to account for ≧50% of all cells when ranked by frequency.

Pearson and Spearman correlations were calculated using scipy.stats.pearsonr and scipy.stats.spearmanr.

ICC was calculated using a two-way random-effects model for absolute agreement as: $ICC\left(2,1\right)=\frac{MS_{B}-MS_{E}}{MS_{B}+\left(k+1\right)MS_{E}}$, where $MS_{B}$ is the mean square between subjects, $MS_{E}$ is the residual mean square, and k is the number of raters (two in this case).

The data was downsampled using scanpy.pp.subsample.

To assess the agreement in TCR clonal diversity profiles between platforms, we calculated Euclidean agreement scores and cosine similarities between matched patient samples. First metrics were min-max scaled to [0,1] using the fit_transform function from sklearn.preproceessing.MinMaxScaler. Second, Euclidean distance was calculated using scipy.spatial.distances.euclidean and transformed into Euclidean agreement score as: $Eucledian\;Agreement=\frac{1}{1+d_{E}}$ where $d_{E}$ is the Euclidean distance. Cosine similarity was computed using sklearn.metrics.pairwise.cosine_similarity.

### 2.7 Statistical analysis and data visualisation

All statistical analyses were performed in Python using scipy.stats. Visualisation was performed in Python (v3.12.7) using seaborn (v0.13.2) and matplotlib (v3.9.2), unless otherwise stated.

## 3 Results

### 3.1 Patients, biological samples, and molecular data generation

Blood samples were collected from seven patients with bladder cancer during routine clinical visits at Aarhus University Hospital. The cohort included five males and two females, aged 65 to 87 years, representing both muscle-invasive and non-muscle-invasive bladder cancer. These clinical variables were not used as stratification factors in the present analysis, as the aim of this work was to benchmark single-cell platforms rather than investigate disease-associated immune variation.

**Fig. 1:**
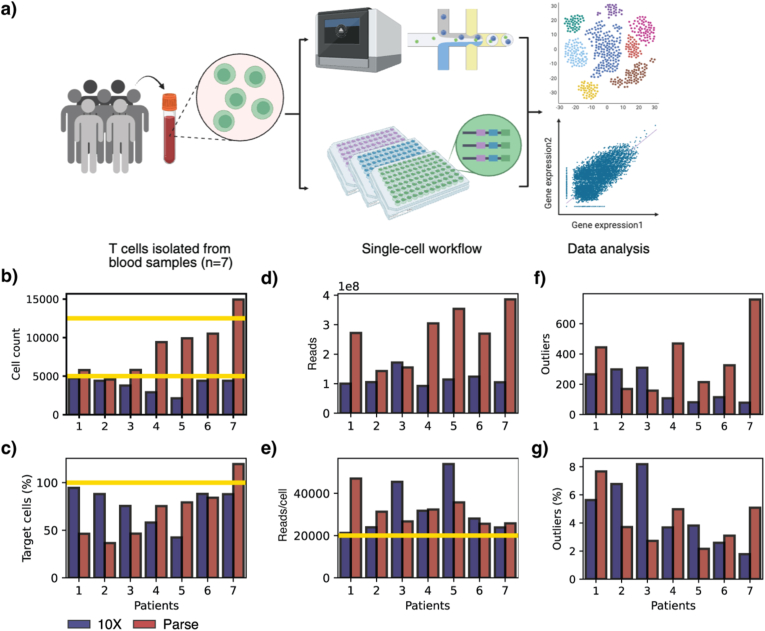
a) Illustration of the workflow. Created with Biorender. b)Barplot showing the cell recovery per sample. Yellow lines indicate cell target, 5000 for 10X and 12500 for Parse. c) Barplot showing the cell recovery per sample as percentage of the target. Yellow line indicates 100% recovery. d)Barplot showing read count per sample. e) Barplot showing reads per cell per sample. Yellow line indicates recommended minimum from the manufacturers, 20.000. f) Barplot showing number of cells labelled as outliers per sample. g) Barplot showing the percentage of cells labelled as outliers per sample.

Following an immediate transfer to the laboratory, T cells were isolated from each sample and assessed for viability. Mean cell viability was 99.6% (range: 98.2 – 100%). Subsequently, cell volumes corresponding to the target recovery were allocated to the Chromium Next GEM Single Cell 5’ Reagent Kits v2 workflow (10X Genomics), while the remaining cell suspensions were fixed and processed using the Evercode TCR workflows (Parse Biosciences; Fig. 1a).

### 3.2 Raw data processing and quality assessment show high sample quality despite variable cell recovery

We aimed to recover 5000 cells per sample for 10X and targeted 12,500 cells per sample for Parse. Both protocols recommend a minimum sequencing depth of 20,000 read pairs per cell, corresponding to target read counts of 1 × 10^8^ per 10X sample and 2.5 × 10^8^ per Parse sublibrary. This target was met for all 10X samples, whereas 5/8 Parse sublibraries fell slightly below this threshold (2.484 × 10^8^ ± 0.172 × 10^8^; Supplementary Fig. 1a).

The data was pre-processed using CellRanger (10X) and split-pipe (Parse), which includes initial read filtering. Across all libraries, approximately 88% of raw reads contained a valid barcode, indicating low background noise (10X: 82.92–90.75%; Parse: 86.92–88.66%; Supplementary Fig. 1b). Of these, ∼71% (10X: 61.97–77.09%) and ∼65% (Parse: 64.51–66.72%) were successfully mapped to the transcriptome (Supplementary Fig. 1c).

Sequencing saturation was generally higher for 10X (62.44–96.42%) but more consistent across samples for Parse (54.91–63.33%; Supplementary Fig. 1d). Actual cell recovery ranged from 2123 to 4725 (42.5 - 94.48% of the target) for 10X and 4563 to 14,939 (36.5 -119.5%) for Parse (Fig. 1b and c).

This resulted in final read counts per sample ranging from 0.9 × 10^8^ to 1.7 × 10^8^ for 10X and from 1.4 × 10^8^ to 3.9 × 10^8^ for Parse (Fig. 1d). Importantly, all samples exceeded the recommended threshold of 20,000 reads per cell (Fig. 1e).

Next, we assessed cell quality, analysing the 10X samples individually and the Parse samples collectively, as advised by the respective manufacturers. Cells were flagged as outliers and excluded if any of the following metrics deviated by more than five median absolute deviations from the median: total number of counts, number of detected transcripts, or the proportion of reads mapping to the top 20 most highly expressed transcripts. The distribution of these metrics varied among patients in the 10X data, while the Parse data was more uniform (Supplementary Fig. 1e–j). Additionally, cells with >10% of reads mapping to mitochondrial DNA were also excluded (Supplementary Fig. 1k–l).

Overall, approximately 4% of all cells were removed as outliers (10X = 4.7%, Parse = 4.2%), resulting in a dataset of 25,479 cells for 10X and 58,396 cells for the Parse dataset. In both datasets, the number and percentage of outliers varied substantially among patients (Fig. 1f and g), and did not differ significantly between platforms (Wilcoxon, p = 0.9375).

### 3.3 Transcriptome coverage and determination of biologically informative transcripts vary between platforms

**Fig. 2:**
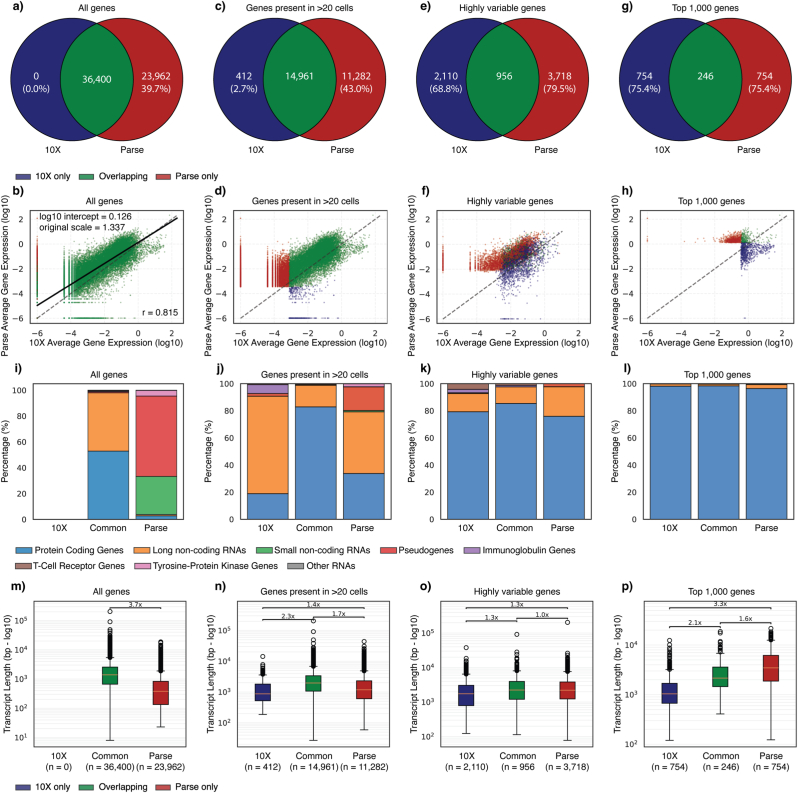
a, c, e, g) Pie chart showing unique and overlapping transcripts when looking at a) all genes, c) genes after filtering those expressed in less than 20 cells, e) genes found to be highly variable, g) top 1000 most highly expressed genes. b, d, f, h) average expression of genes in 10X and Parse when comparing b) all genes, d) genes after filtering those expressed in less than 20 cells, f) genes found to be highly variable, h) top 1000 most highly expressed genes. i-l) percentage of genes annotated as each Ensembl biotype when looking at i) all genes, j) genes after filtering those expressed in less than 20 cells, k) genes found to be highly variable, l) top 1000 most highly expressed genes. m-p) Transcript-length difference between genes unique to either 10X or Parse, or common to both platforms when looking at m) all genes, n) genes after filtering those expressed in less than 20 cells, o) genes found to be highly variable, p) top 1000 most highly expressed genes.

One advantage of the random primers used by Parse Biosciences is their ability to detect a significantly broader range of genes than droplet-based methods, such as those from 10X Genomics. In our comparison, we identified 36,601 genes in the 10X dataset versus 62,710 transcripts in the Parse dataset. Because 10X and Parse preprocessing pipelines use different Ensembl reference versions (Ensembl v98 and v109, respectively), we harmonised the analysis by restricting comparisons to genes present in both reference annotations. After harmonisation, Parse detected all genes observed in the 10X dataset (36,400), as well as an additional 23,962 genes not detected by 10X (Fig. 2a).

Despite this broader detection, average transcript expression between the datasets was highly correlated (Pearson's r = 0.815; Fig. 2b). Linear expression analysis on log-transformed expression values revealed systematically higher expression levels in Parse compared with 10X (log10 intercept = 0.126, corresponding to a 1337-fold increase on the linear scale). This platform-dependent difference was further supported using Bland-Altman analysis, which showed a mean difference of 1.072 log units between Parse and 10X (Supplementary Fig. 2a). However, this global difference was driven mainly by two patients (Patients 6 and 7), suggesting some inter-individual variability (Supplementary Fig. 2b).

To refine our analysis, we removed genes detected in 20 or fewer cells, as such low-frequency transcripts are more likely to reflect stochastic measurement noise than biologically meaningful expression[23]. This filtering step reduced the number of genes by nearly half, down to 15,373 in the 10X dataset (-42.2%) and 26,243 in the Parse dataset (−43.5%). Notably, only 12.5% of the 23,962 Parse-unique genes remained after this filtering step, indicating that the majority were low-abundance and likely less stable or contextually relevant to T cell biology. After filtering, 14,961 genes overlapped between the two datasets (Fig. 2c and d).

To identify biologically informative genes, we applied standard preprocessing pipelines including feature selection based on dispersion and expression levels. We found highly variable genes in each dataset by computing normalised dispersion within bins of similarly expressed genes using log-transformed data. Although both datasets contain tens of thousands of detected genes, only a small subset met the criteria for high variability within each platform. Specifically, 3066 highly variable genes were identified in the 10X dataset and 4674 in the Parse dataset, with 956 overlapping between them (Fig. 2e and f). Similarly, just 246 of the 1000 most highly expressed genes overlapped between platforms (Fig. 2g and h). Notably, more than 97.6% of genes that were highly variable or highly expressed genes in only one dataset were detected by both methods. This suggests that the differences in selected features are not due to one method failing to detect the gene, but rather to platform-specific differences in how gene expression variability is measured and normalised.

To further evaluate the biological relevance of gene discrepancies, we annotated all detected genes by biotypes using Ensembl Release 109 (Fig. 2i–l, Supplementary Table 1). Among the 36,400 genes shared by both platforms, the majority were either protein-coding (19,254; 52.9%) or long non-coding RNAs (lncRNAs; 16,430; 45.1%). Genes unique to Parse were mostly pseudogenes (14,896) and small non-coding RNAs (7,055), with proportionally fewer protein-coding genes (654) and lncRNAs (271; Fig. 2i).

The biotype distribution changed substantially when we only investigated genes present in more than 20 cells (Fig. 2j). Most pseudogenes and small non-coding RNAs were excluded, suggesting that these gene types are less informative for T-cell datasets. Of the 14,961 common genes, 85% (12,392) were protein-coding, and 16% (2395) were lncRNAs. Genes unique to each platform post-filtering were also mostly lncRNAs or protein coding (10X = 296 and 78; Parse = 5122 and 3809). Importantly, the genes labelled as highly variable or among the top 1000 most expressed were mainly protein-coding (Fig. 2k and l).

We next examined whether transcript length differed between platform-specific and shared gene sets (Fig. 2m–p). Across all genes, those unique to Parse had shorter transcripts compared to genes detected by both methods (3.7-fold difference; Fig. 2m), reflecting Parse's broader detection of short genes such as small non-coding RNAs. However, this pattern was strongly dependent on expression and filtering criteria. After restricting the analysis to genes expressed in >20 cells, the difference was substantially reduced (Fig. 2n), and among highly variable genes, transcript lengths were comparable across platform-specific and shared gene sets (Fig. 2o). Notably, within the top 1000 most highly expressed genes, Parse-unique genes had longer transcripts than genes unique to 10X (3.3-fold difference; Fig. 2p), indicating that transcript length bias becomes more apparent among highly expressed genes.

In summary, Parse captures a broader and more diverse transcriptome, including many pseudogenes and small non-coding RNAs. However, most of these were filtered out due to low abundance or limited expression across cells, and downstream analyses were primarily driven by protein-coding genes detected by both methods. Yet, due to platform-specific length biases and differences in how expression variability is measured, the overlap in selected transcripts was limited. Rather than indicating that one platform selects the ‘wrong’ genes, this highlights that biologically meaningful variation may be captured differently across platforms. As a result, cross-platform comparisons should be interpreted with caution, especially when relying on variable gene selection.

### 3.4 Protocol design impacts batch effect and performance of batch correction

Single-cell data is prone to batch effects introduced during sample collection or processing. To assess the batch effect in our data, we utilised a single-cell variational inference tool (scVI), a package for probabilistic deep learning tools, using the patient identity specified as the batch variable.

The training performance differed notably between the two datasets, reflecting their inherent differences. For the 10X dataset, scVI completed 111 epochs and achieved a final validation loss of 4476.5, while for Parse, the model converged after 86 epochs with a higher final loss of 8163. The initial loss values also differed (∼6000 for 10X and ∼9200 for Parse), likely due to the larger number of cells and transcripts in Parse. Despite these differences, both datasets showed smooth loss curves across epochs, indicating stable model training (Supplementary Fig. 3a).

**Fig. 3:**
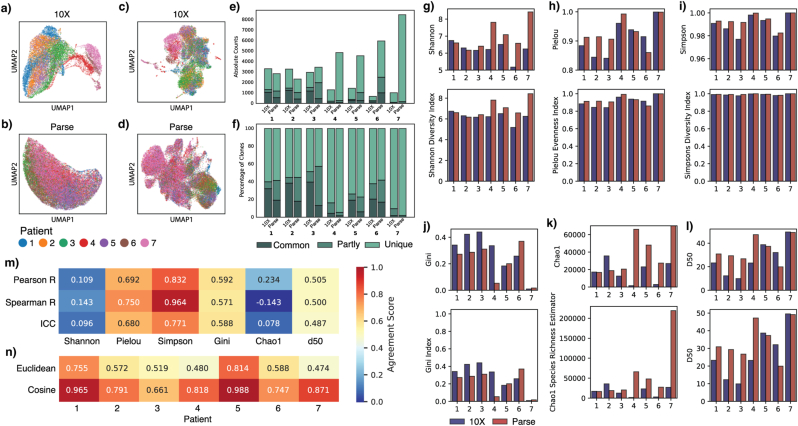
a-d) UMAPs coloured by patients from a) 10X before batch correction, b) Parse before batch correction, c) 10X after batch correction with scVI, d) Parse after batch correction with scVI. e-f) clones overlapping between 10X and Parse, partly overlapping or unique to each method, shown as e) absolute clone count and f) percentage of clones. g-l) Diversity of each sample calculated using g) Shannon Diversity Index, h) Pielou Evenness Index, i) Simpsons Diversity Index, j) Gini Index, k) Chao Species Richness Estimator, and l) D50. Lower panel shows whole scale from 0, and upper panel highlights the changes. m) Correlation calculations between 10X and Parse for each diversity index. n) Euclidean agreement and Cosine similarity of each diversity index for each patient.

We then visualised the batch effect using uniform manifold approximation and projection (UMAP). Before batch correction, UMAP was computed on a nearest-neighbour graph of a principal component analysis (PCA) representation of the data, using the first 30 principal components (Fig. 3a and b). In this space, the 10X data showed a clear separation by patients, while the Parse data showed a more intermixed structure.

After applying scVI-based batch corrections, we generated new UMAPs computed on a nearest-neighbour graph constructed with the scVI latent representation of the data (Fig. 3c and d). Both datasets demonstrated improved integration, supporting the effectiveness of scVI in mitigating batch effects despite initial structural differences.

The intermixed structure may have contributed to scVI's lower performance on the Parse data, as weaker sample-associated separations can make it more challenging for the model to learn batch-specific corrections.

Taken together, this highlights that platform-specific design can influence the apparent structure of single-cell embeddings in UMAP space.

### 3.5 TCR repertoire analysis reveals variation in clone recovery and diversity across methods

Both platforms showed comparable TCR recovery, with detectable TCR alpha (TRA) or beta (TRB) chains in 55.1% of 10X cells (14,036 of 25,479) and 55.8% of Parse cells (32,579 of 58,396). Notably, one in eight TCR sublibraries from Parse failed during library preparation, yet the overall recovery was similar to that of 10X. This indicates that Parse may have a slightly higher TCR recovery efficiency despite the incomplete data.

To compare clonal repertoires between the two platforms, we defined TCR clones based on the paired alpha and beta chains within each patient. Clones were categorised as overlapping if the same TRA-TRB pair was present in both patient samples, partly overlapping if some chains were overlapping but not both as a pair, and unique if both chains were found in only one of the patient samples. This revealed substantial variation between the patients, with some repertoires exhibiting significant overlap and others showing minimal overlap (Fig. 3e and f, Supplementary Fig. 3b). Importantly, more than 95% of the unique clones consisted of a single cell, except for sample 5 from 10X, where only 68% of the clones were single-cell clones. This indicates that both methods provide comparable representations of the TCR landscape, as rare clones like these are unlikely to appear in both datasets due to stochastic sampling rather than methodological differences.

We analysed the diversity of the TCR repertoire using multiple commonly utilised diversity indices, including the Shannon Diversity Index, the Pielou Evenness Index, the Simpson's Diversity Index, the Gini Index, the Chao1 Richness Estimator, and the D50 Index (Fig. 3g–l). These indices capture different aspects of TCR diversity: richness, evenness, and dominance. To evaluate platform agreement, we compared the diversity metrics derived from 10X and Parse individually and in combination through multi-dimensional analyses (Fig. 3m and n, Supplementary Table 2).

We found that diversity metrics sensitive to rare clonotypes, specifically Shannon and Chao1, showed the lowest platform agreement, with low and non-significant correlations and an intraclass correlation coefficient (ICC). Shannon integrates species richness and evenness, performing best when clonal distribution is not highly skewed. Chao1estimates the number of ‘missing’ clonotypes based on rare species abundance patterns and is most informative in datasets rich in low-abundance clones. To determine whether these discrepancies were caused by the differences in cell capture, we downsampled the Parse dataset to match 10X cell counts and recalculated both metrics (Supplementary Fig. 3c). Although the diversity was slightly lower in the downsampled data, the divergence between the platforms remained. This indicates that metrics sensitive to rare clonotypes are substantially influenced by platform-specific differences in cell recovery and detection efficiency, independent of overall sampling depth.

Metrics emphasising evenness, Pieloue and D50 showed moderate but non-significant correlations and ICC values. Pielou, the normalised form of Shannon, measures uniformity in clonal distribution. D50 measures clonal skew by calculating the number of clones needed to account for 50% of cells, starting from the most abundant. Low D50 values indicate dominance by a few clones, while higher values suggest more even distributions. These results indicate that although evenness patterns are partially preserved across platforms, absolute values remain sensitive to differences in sampling depth and cell recovery.

In contrast, methods emphasising clonal dominance, Simpson's and Gini, demonstrated stronger inter-platform agreement. The Simpsons achieved the highest concordance, with statistically significant correlations (Pearson r = 0.832, p = 0.020 and Spearman r = 0.964, p < 0.001) and a strong ICC value (ICC = 0.771). By calculating the probability that two randomly chosen T cells belong to the same clonotype, Simpson's is inherently less affected by rare clones, making it more robust to technical variability such as inconsistent sampling depth or cell recovery efficiency. Notably, it was the only metric that showed a significant difference between platforms, with Parse scoring 0.178 points higher on average (p = 0.016). This reflects Parse's ability to recover not just rare but also mid-frequency clonotypes, thereby improving overall clonal evenness and elevating its Simpson values. Gini measures inequality among clone frequencies, showing only moderate and non-significant correlations and ICC, and appears more sensitive to platform-specific differences.

To assess overall agreement in diversity profiles, we calculated Euclidean agreement and cosine similarity across all six normalised metrics for each patient (Fig. 3n). Euclidean agreement (inverse Euclidean distance) reflects the overall similarity in absolute diversity values. In contrast, cosine similarity captures the similarity in the pattern or shape of the diversity profiles, regardless of magnitude. Higher values for both indicate greater concordance between platforms. This multidimensional approach revealed substantial inter-patient variability: Patient 5 showed excellent agreement (Euclidean agreement = 0.816, cosine similarity = 0.991), whereas Patients 3 and 6 displayed notable discordance (Euclidean agreements = 0.496 and 0.547; cosine similarities = 0.624 and 0.688, respectively). Nevertheless, the median cosine similarity across all patients was 0.867, indicating good pattern agreement despite absolute value differences, suggesting that both platforms capture similar underlying TCR diversity structures.

In summary, platform consistency varied systematically based on the sensitivity of each metric to sampling depth and rare clonal detection. While both platforms captured major clonal populations and comparable TCR diversity patterns, Parse's higher cell recovery consistently elevated metrics dependent on the detection of rare clones. Our data suggests that the Simpsons Diversity Index is the most robust choice for cross-platform comparisons. However, cell-recovery-related sampling effects must still be accounted for, particularly in meta-analyses or when drawing biological conclusions about rare clonotype populations.

### 3.6 Gene detection biases influence the interpretation of T cell subtypes

**Fig. 4:**
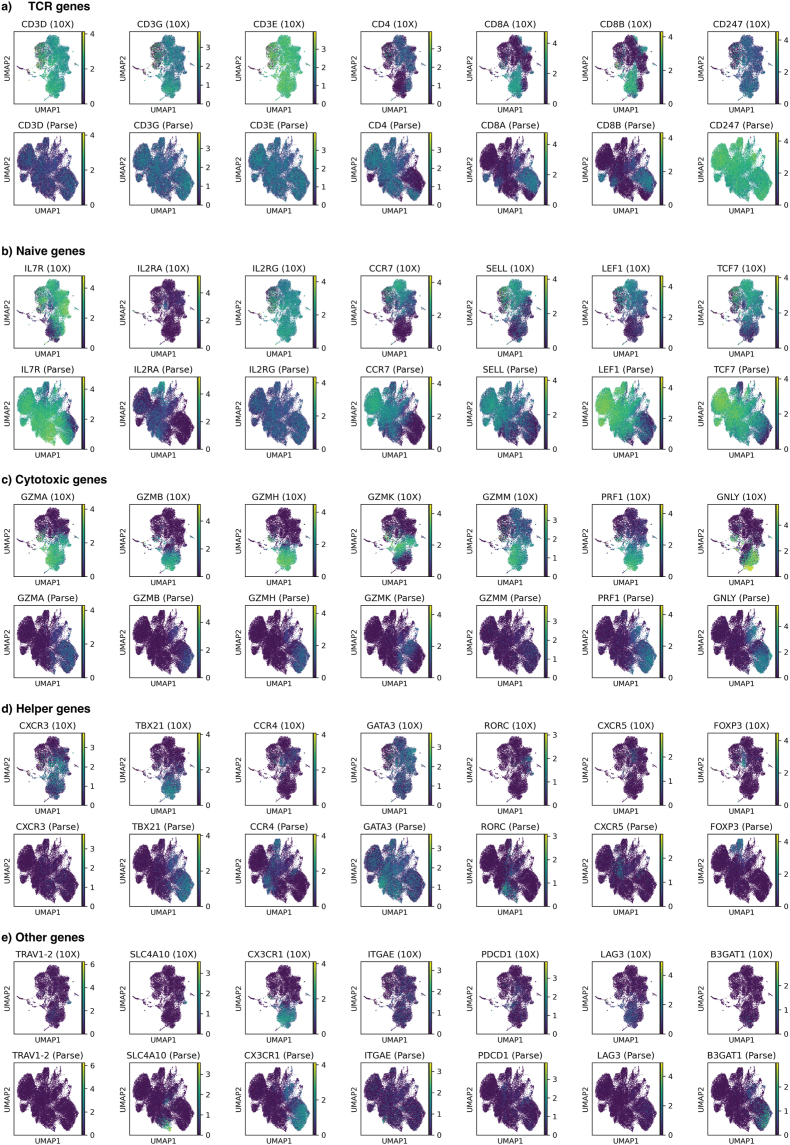
UMAPs coloured by gene expressions for a) TCR complex, b) Naive T cells, c) Cytotoxic T cells, d) Helper T cells, e) MAIT cells (TRAV1-2 and SLC4A10), Temra cells (CX3CR1), Trm (ITGAE), exhaustion markers (PDCD1 and LAG3) senescence marker (B3GAT1). 10X in the upper panel and Parse in the lower panel.

We inspected several genes typically used to define T cell subtypes, starting with those comprising the TCR complex (*CD3D, CD3G, CD3E, CD247, CD4, CD8A,* and *CD8B*; Fig. 4a, Supplementary Table 3). Since the data was filtered based on the presence of a TCR, we expected to see an expression of all *CDR3* genes and either *CD4* or *CD8A/B*. However, we observed a dramatic difference in the detection rates across platforms. 10X detected CD3 genes in a higher proportion of cells and demonstrated a 3 to 5.7x higher mean expression, indicating a superior sensitivity for core T cell markers. Interestingly, Parse detected *CD247* (CD3-zeta) more frequently. Of the lineage markers, CD4 was better captured by Parse, whereas CD8A and CD8B were better captured by 10X. These discrepancies could impact downstream analysis, but CD4 and CD8A/B marked different cell populations in both datasets, making them less worrisome. Notably, a double-negative population was observed in the 10X dataset, which was not apparent in the Parse dataset.

We next examined genes associated with naive and central memory T cells (Tcm; *IL7R, IL2RG, IL2RA* (negative marker)*, CCR7, SELL, LEF1,* and *TCF7*; Fig. 4b–Supplementary Table 3). Parse exhibited higher expression of most markers and found them in more cells, while 10X showed higher mean expression of *IL2RG* and *SELL*. Generally, there was a good agreement on the coexpression, with some differences indicating different states.

To analyse helper T cell subsets, we inspected markers for Th1 (*CXCR3* and *TBX21*), Th2 (*CCR4* and *GATA3*), Th17 (*RORC*), Tfh (*CXCR5*), and Treg (*FOXP3*; Fig. 4c–Supplementary Table 3). *CXCR3* showed a striking 14.7x higher expression in 10X, whereas the other markers were more balanced between platforms.

Then, we investigated cytotoxic potential by analysing cytotoxic proteins: granzymes (*GZMA*, *GZMB*, *GZMH*, *GZMK,* and *GZMM*), perforin (*PRF1*), and granulysin (*GNLY*; Fig. 4d–Supplementary Table 3). 10X exhibited strikingly higher detection and expression of all granzymes and perforin, with granzymes showing 7-17x higher expression. These genes were generally found to be highly expressed in cells lacking naive transcript expression, indicating a robust cytotoxic signature of active and differentiated cytotoxic cells. Intriguingly, we identified populations of CD4^+^ T cells with cytotoxic features in both datasets, which have been linked with anti-tumour activity.

Lastly, we examined gene characterising MAIT cells (*TRAV1-2* and *SLC4A10*), Temra cells (*CX3CR1*), and Trm (*ITGAE*), along with exhaustion markers (*PDCD1* and *LAG3*) and a senescence marker (*B3GAT1*; Fig. 4e–Supplementary Table 3). There was good agreement between the expression of *TRAV1-2* and *SLC4A10* in the 10X dataset. However, while *TRAV1-2* was close to undetected by Parse, *SLC4A10* was present in a distinct population. The Temra and Trm markers were comparable between the methods, but 10X detected more exhaustion, while Parse detected more senescence.

We observed a significant negative correlation between transcript length and expression difference across subtype-specific genes (Supplementary Fig. 3d; Spearman rho = −0.44, p = 0.008). 10X preferentially detects shorter transcripts at higher levels, whereas Parse shows better relative detection of longer transcripts, consistent with the transcript-length biases identified in the broader gene-set analysis.

These gene detection biases may influence how T cell subtypes are computationally resolved, underscoring the importance of carefully choosing markers across platforms.

## 4 Discussion

This manuscript compares two methods for single-cell paired TCR sequencing with whole transcriptome profiling, 10X Genomics and Parse Biosciences. Utilising matched patient samples, we assessed transcriptome coverage, batch effects, TCR clonal recovery and diversity, and the detection of genes relevant for T cell subtype identification. To fully assess each platform's capabilities, we analysed the data at their natural throughput without downsampling, thereby reflecting real-world scenarios. While different cell recovery targets were set for each platform, both were within the manufacturer's recommended range and reflect realistic experimental conditions. As our comparison focuses on data quality and transcriptomic features rather than absolute cell numbers, these differences are not expected to affect the overall conclusion. Still, future studies may consider matching cell recovery targets to enable more direct platform comparisons. The relatively small cohort size limits the statistical generalisability of our findings but provides a controlled, sample-matched design. Future large-cohort studies could build on these benchmarking results to validate the platform-specific biases identified here and further characterise the conditions under which one platform may be favored over the other. We followed the Single-cell best practices [23] for the analysis while incorporating manufacturer recommendations where applicable.

The library preparation protocols for both methods are extensive and require multiple days of laboratory work [19,21]. Both platforms incorporate safe stopping points, where samples can be stored cold or frozen for extended periods, providing flexibility in experimental planning. The Parse protocol starts by fixating and permeabilising the cells, making them stable for up to six months. This provides additional flexibility and scalability for sample collection, which can be pivotal for time-course studies. Collected samples are mixed and processed simultaneously, minimising the time spent on library preparation. This means that even with a longer protocol (approximately 23 h), if many samples are collected over extended timelines, Parse can be logistically more efficient than 10X (approximately 14.5 h). For complex study designs such as multi-timepoint or large cohort experiments, Parse may be the preferred choice, while 10X's live-cell workflow may be more straightforward for small-scale studies. Notably, while 10X recently released a fixation protocol for GEM-X Assays [28], enabling both 3′ and 5′ capture on fixed cells, it was not available when the samples in this study were processed, and it remains incompatible with the protocol used here.

The premixing of samples in the Parse protocol is also expected to reduce technical batch effects arising from independent sample processing, consistent with the more intermixed structure observed in the UMAP embeddings. In contrast, the 10X data exhibited clear patient-specific separation. However, an intermixed structure does not necessarily imply improved biological integration, as true patient-specific transcriptional programs may also be less apparent depending on platform-specific capture characteristics. Interestingly, a previous study reported the opposite when using the 10X's 3′ chemistry, where Parse showed stronger batch effects [18]. These conflicting observations may be due to differences in cDNA synthesis between 10X's 3′ and 5′ methods, but they highlight that relative batch behaviour is context-dependent and can be influenced by both chemistry and experimental design.

These differences in chemistry not only affect batch effects but also underlie distinct cDNA synthesis strategies, which in turn influence transcriptome coverage, bias, and efficiency. 10X uses primers that bind to the poly(A) tail of mRNA transcripts and initiate reverse transcription from the 3′ end. When the reverse transcription reaches the 5′ end of the mRNA, it naturally adds extra nucleotides (typically C residues) which are recognised and bound to by template-switching oligos. RT then continues extending the cDNA using the TSO as a new template, ensuring the inclusion of the true 5′ end of the transcript. However, this method is sensitive to RNA degradation and restricted to polyadenylated transcripts, but captured transcripts are unbiased in coverage, and full-length V(D)J sequences are ensured. Parse utilises a combination of oligo-dT and random hexamer primers, allowing broader transcript coverage and not restricted to polyadenylated RNAs [29]. While this method is more resilient to RNA degradation [15], it does not guarantee full-length capture, depending on the binding site of the random primers. Consequently, some transcripts may lack their true 5’ end, and full V(D)J sequence coverage cannot be guaranteed.

Despite these distinct cDNA synthesis strategies, TCR chain recovery and clonal diversity were only minimally affected, a finding not previously reported. Both methods captured at least one TCR chain (TRA and/or TRB) in approximately 55% of recovered cells. One of Parse's eight TCR sublibraries failed during library preparation, for unknown reasons, corresponding to an estimated 12.5% loss in capture efficiency. Importantly, as Parse sublibraries represent pooled mixtures of all patients rather than sample-specific libraries, this loss was distributed across the cohort instead of affecting any individual patient disproportionately. TCR sequences recovered from the remaining sublibraries were therefore carried forward into analysis without further adjustment. Still, a higher capture rate might have been expected from 10X, where no libraries failed. Nonetheless, both methods provided comparable representations of the overall TCR landscape and reliably detected dominant TCR clones. Accordingly, metrics capturing clonal dominance patterns (Simpson's D and Pielou's evenness) showed superior cross-platform agreement. In contrast, metrics sensitive to rare clones (Shannon, Chao1, and D50) showed poor concordance, likely due to Parse's stronger recovery as reflected by its higher Simpson's D. Finally, although absolute diversity values differed between platforms, cosine similarity analyses indicated that overall clonal diversity patterns were largely preserved. This highlights the importance of selecting appropriate diversity metrics that align with the specific research question and underscores the value of multi-metric assessments for obtaining robust, biologically meaningful insights.

Consistent with prior comparisons between Parse and 10X 3′ platforms [18], we found that Parse detected more genes than the 10X 5′ system. This aligns with the broader capture range expected from Parse's combined oligo(dT) and random hexamer priming strategy, compared with the oligo(dT)-only priming used by 10X. However, our analyses revealed that the increased gene yield was not uniform across gene classes and was strongly shaped by transcript-length bias in a gene-set-dependent manner.

Both platforms detected protein-coding genes and lncRNAs, but most Parse-specific genes fell into shorter non-coding categories. Historically, these were often dismissed as non-functional byproducts of RNA processing, but recent studies indicate that they have biological relevance. Pseudogenes, comprising the largest part of the Parse-specific genes, are defective gene copies that retain sequence similarities to functional genes but harbour frameshifts and premature stop codons. While their functions are not well understood, some pseudogenes are known to influence gene regulation by generating small interfering RNAs or changing the expression of protein-coding genes [30]. Another class enriched among Parse-specific genes was sncRNAs. These include diverse genes involved in RNA processing, gene silencing, and translational control [24,30]. However, the functional relevance of both pseudogenes and sncRNAs is heavily context dependent. Their inclusion in transcriptome analyses can introduce noise or complicate interpretation. After applying our ≥20-cell expression filter, nearly all pseudogenes and sncRNAs were removed, indicating that they are typically low-abundance in T cells. This suggests they are unlikely to drive the primary biological signals in T-cell transcriptome analyses, meaning that Parse's broader capture range is not necessarily advantageous in this context. Since immunophenotyping and functional subtyping are primarily based on protein-coding effector and signalling genes, which were rarely unique to Parse, the additional genes captured by Parse are unlikely to improve subtype resolution.

The transcript-length bias was most pronounced within the top 1000 most highly expressed genes, where the overlap between platforms was minimal. Here, the Parse dataset was enriched for longer transcripts, while the 10X dataset was enriched for shorter transcripts. This has biological implications relevant for T cell studies, as cytotoxic and effector transcripts tend to be shorter. These were more efficiently detected by 10X, making it well-suited for studies focused on effector function. However, Parse's broader coverage and ability to detect longer transcripts may be advantageous for exploratory analyses of transcriptomic heterogeneity, particularly in naive or less-activated T cell populations, where subtle regulatory differences rather than effector programs are of primary interest [17,24] Consistent with previous findings [17,18], 10X was also superior at capturing genes comprising the TCR complex, likely due to the transcript-length bias, as these genes are generally short. These findings emphasise the importance of accounting for platform-specific sequence capture biases when choosing marker transcripts and interpreting T cell heterogeneity and function across single-cell technologies.

A double-negative T cell population was observed exclusively in the 10X dataset, which may reflect either a true biological population captured with greater sensitivity by 10X's cDNA synthesis chemistry, or a technical artefact such as ambient RNA contamination, which is a known limitation of droplet-based capture systems. Definitive resolution would require further validation in larger cohorts.

In summary, both 10X Genomics and Parse Biosciences platforms enable effective single-cell TCR and transcriptome profiling but differ in key technical and logistical aspects. 10X provides more consistent full-length TCR capture and strong recovery of shorter transcripts, while Parse offers broader transcriptomic coverage, and greater flexibility through fixation. These differences represent trade-offs rather than a clear hierarchy, underscoring the importance of matching platform strengths with study objectives, whether the priority is clonal resolution, transcriptomic breadth, or experimental design constraints. That will ultimately yield the most accurate and biologically meaningful insights.

## Declaration of generative AI and AI-assisted technologies in the manuscript preparation process

During the preparation of this work, the author(s) utilised ChatGPT and Claude.ai for coding assistance, as well as ChatGPT and Grammarly for writing and language refinement. After using these tools, the author(s) reviewed and edited the content as needed and take full responsibility for the content of the published article.

## Funding

This work was supported by Aarhus University and Aarhus University Hospital.

## CRediT authorship contribution statement

**Nanna Kristjánsdóttir:** Conceptualization, Data curation, Formal analysis, Investigation, Visualization, Writing – original draft, Writing – review & editing. **Kasper Thorsen:** Conceptualization, Investigation, Writing – review & editing. **Nicolai J. Birkbak:** Writing – review & editing. **Lars Dyrskjøt:** Conceptualization, Funding acquisition, Resources, Writing – review & editing.

## Declaration of competing interest

The authors declare the following financial interests/personal relationships which may be considered as potential competing interests:Nanna Kristjansdottir reports financial support was provided by C2I Genomics, Inc. Nanna Kristjansdottir reports financial support was provided by Novo Nordisk Foundation. Lars Dyrskjot reports a relationship with C2I Genomics, Inc. that includes: funding grants. Lars Dyrskjot reports a relationship with Natera, Inc. that includes: funding grants. Lars Dyrskjot reports a relationship with AstraZeneca that includes: funding grants and speaking and lecture fees. Lars Dyrskjot reports a relationship with Photocure that includes: funding grants. Lars Dyrskjot reports a relationship with Ferring that includes: consulting or advisory. Lars Dyrskjot reports a relationship with MSD that includes: consulting or advisory and travel reimbursement. Lars Dyrskjot reports a relationship with Cystotech that includes: consulting or advisory. Lars Dyrskjot reports a relationship with UroGen that includes: consulting or advisory. Lars Dyrskjot reports a relationship with Pfizer that includes: speaking and lecture fees. Lars Dyrskjot reports a relationship with Roche that includes: consulting or advisory. Lars Dyrskjot reports a relationship with BioXpedia that includes: board membership. If there are other authors, they declare that they have no known competing financial interests or personal relationships that could have appeared to influence the work reported in this paper.

## Data Availability

Due to Danish legislative requirements and the terms of patient consent, sensitive and pseudonymized data cannot be made publicly available. All analysis code is publicly available through GitHub: https://github.com/nankrj/scRNA_TCRseq_platform_comparison
