# Supplementary material for: Immune profiling of T cells: A comparative analysis of single-cell TCR sequencing using 10X Genomics and Parse Biosciences platforms
Source: Biochem Biophys Rep. 2026 Apr 19;46:102592. doi: 10.1016/j.bbrep.2026.102592 (PMC13103580; doi:10.1016/j.bbrep.2026.102592)
Supplement: Multimedia component 1 [file mmc1.docx]

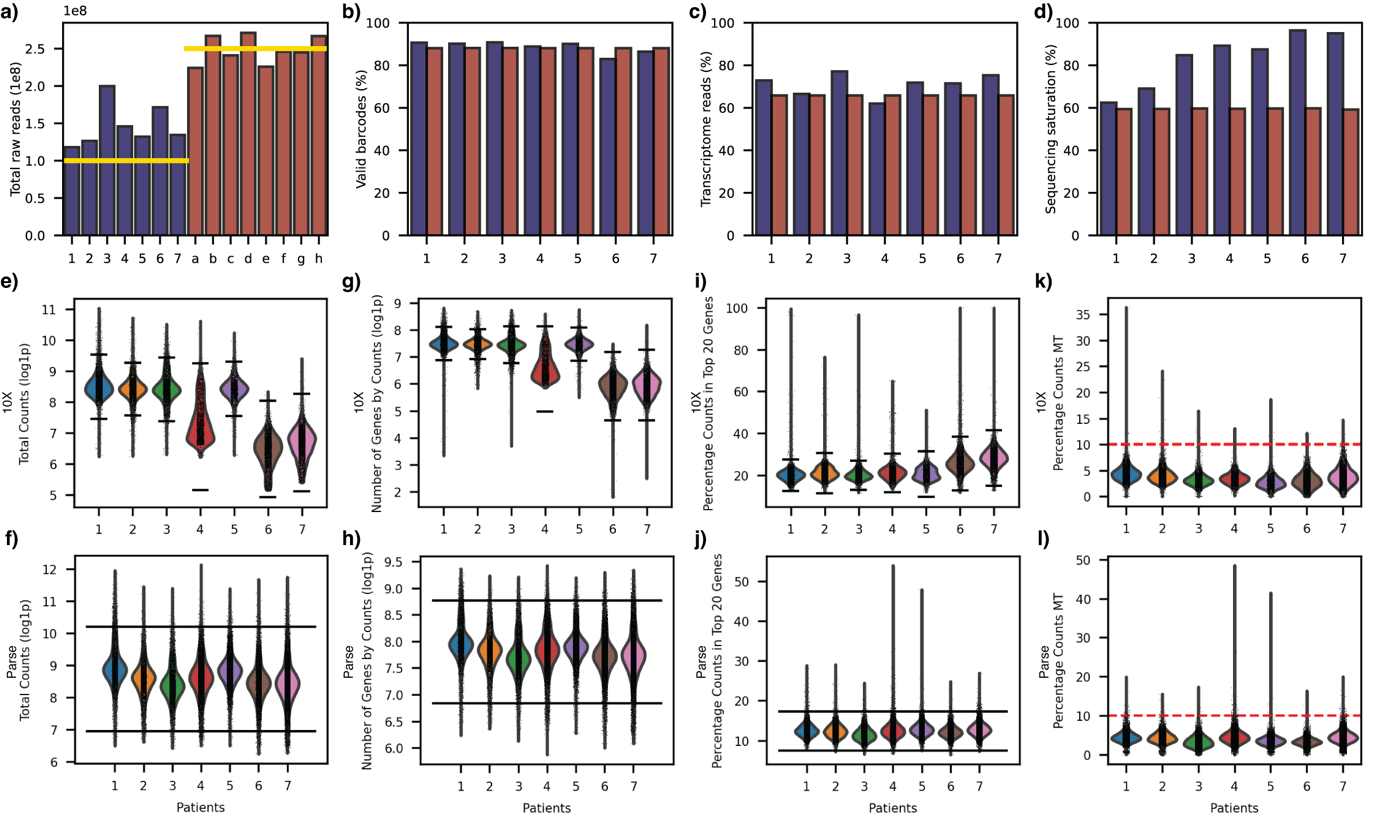


**Supplementary Figure 1**

a) Barplot showing total raw reads per 10X sample and Parse sublibrary. Yellow line indicate read target based on manufacturer recommendation for reads per cell. b) Barplot showing percentage of reads with valid barcodes. c) Barplot showing percentage of reads mapped to the transcriptome. d) Barplot showing sequencing saturation. e-l) Violin plots showing QC measures used to identify outliers in e, g, i, k) the 10X dataset, and f, h, j, l) the Parse dataset.


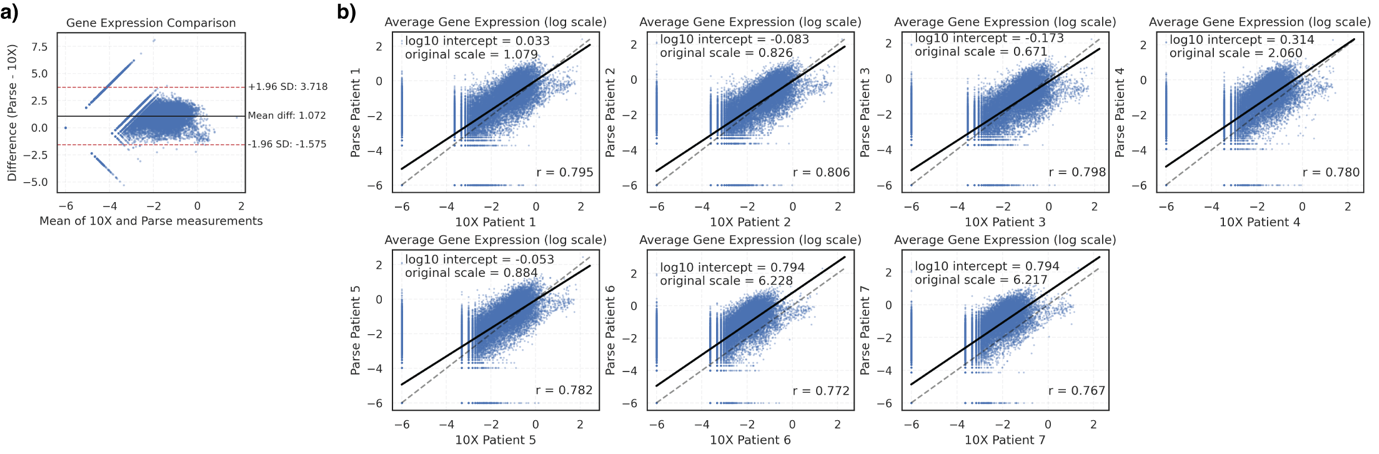


**Supplementary Figure 2**

a) Bland-Altman analysis of average transcript expression between 10X and Parse. b) Correlation of average transcript expression between 10X and Parse for each patient individually.


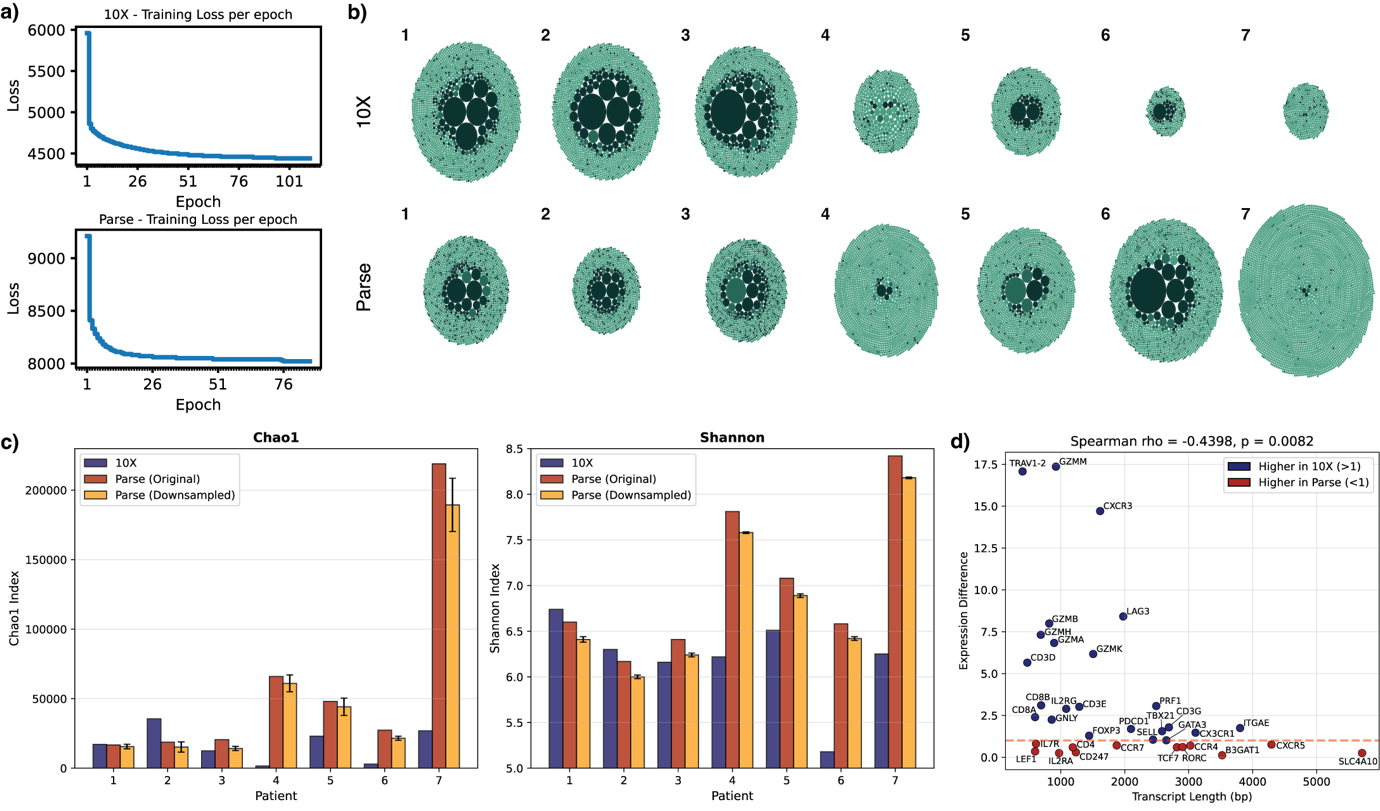


**Supplementary Figure 3**

a) Training Loss per epoch during batch correction using scVI for 10X (upper panel) and Parse (lower panel). b) Visualisation of clones in each sample, coloured by overlap. c) Chao1 and Shannon diversity calculated with natural throughput of 10X and Parse, and downsampled Parse data. The bars show the mean of 10 downsamples, and the error bars show the standard deviation. d) Spearman correlation between average gene expression differences between 10X and Parse, and transcript length.

|  |  | **Protein coding** | **lncRNA** | **sncRNA** | **pseudogene** | **IGX gene** | **TRX gene** | **TEC** | **Other RNAs** |
| --- | --- | --- | --- | --- | --- | --- | --- | --- | --- |
| **All genes** | Common | 19,254 | 16,430 | 0 | 298 | 214 | 195 | 1 | 17 |
|  | 10X | 0 | 0 | 0 | 0 | 0 | 0 | 0 | 0 |
|  | Parse | 654 | 271 | 7,055 | 14,896 | 0 | 0 | 1050 | 36 |
| **>20 cells** | Common | 12,392 | 2,395 | 0 | 24 | 45 | 100 | 0 | 5 |
|  | 10X | 78 | 296 | 0 | 8 | 27 | 3 | 0 | 0 |
|  | Parse | 3,809 | 5,122 | 110 | 1,970 | 7 | 4 | 244 | 16 |
| **Highly variable** | Common | 816 | 117 | 0 | 0 | 11 | 12 | 0 | 0 |
|  | 10X | 1671 | 286 | 0 | 11 | 54 | 87 | 0 | 1 |
|  | Parse | 2,821 | 810 | 4 | 82 | 0 | 0 | 0 | 1 |
| **Top 1000** | Common | 242 | 3 | 0 | 0 | 0 | 1 | 0 | 0 |
|  | 10X | 740 | 12 | 0 | 0 | 0 | 2 | 0 | 0 |
|  | Parse | 727 | 22 | 2 | 1 | 0 | 0 | 0 | 2 |

**Supplementary Table 1: Overview of number of transcripts in each biotype.**

Numbers behind figure 2i.

| **Measure** | **Pearson r (p-value)** | **Spearman r (p-value)** | **ICC** | **Wilxocon (p-value)** | **Agreement** |
| --- | --- | --- | --- | --- | --- |
| **Shannon** | 0.109 (0.816) | 0.143 (0.760) | 0.096 | 0.078 | Poor |
| **Pielou** | 0.692 (0.085) | 0.750 (0.052) | 0.680 | 0.375 | Moderate |
| **Simpson** | 0.832 (0.020) | 0.964 (<0.001) | 0.771 | 0.016 | Strong |
| **Gini** | 0.592 (0.161) | 0.571 (0.180) | 0.588 | 0.297 | Moderate |
| **Chao1** | 0.234 (0.613) | -0.143 (0.760) | 0.078 | 0.109 | Poor |
| **d50** | 0.505 (0.247) | 0.500 (0.253) | 0.487 | 0.297 | Moderate |

**Supplementary Table 2: Overview of measures for each diversity index.**

Numbers behind figure 3m.

| **Gene** | **Nonzero %** | | | **Mean** | | | **Std Dev** | | |
| --- | --- | --- | --- | --- | --- | --- | --- | --- | --- |
|  | **10X** | **Parse** | **Difference** | **10X** | **Parse** | **Ratio** | **10X** | **Parse** | **Ratio** |
| **CD3D** | 84.433 | 28.356 | 56.077 | 1.711 | 0.303 | 5.654 | 0.887 | 0.518 | 1.710 |
| **CD3G** | 64.244 | 48.258 | 15.986 | 1.039 | 0.580 | 1.791 | 0.873 | 0.661 | 1.320 |
| **CD3E** | 91.163 | 58.040 | 33.123 | 2.220 | 0.736 | 3.017 | 0.823 | 0.697 | 1.181 |
| **CD247** | 48.183 | 96.345 | -48.161 | 0.700 | 2.353 | 0.298 | 0.791 | 0.737 | 1.074 |
| **CD4** | 22.619 | 40.177 | -17.558 | 0.296 | 0.499 | 0.594 | 0.583 | 0.667 | 0.874 |
| **CD8A** | 29.965 | 19.530 | 10.435 | 0.618 | 0.258 | 2.400 | 0.994 | 0.564 | 1.763 |
| **CD8B** | 29.642 | 16.112 | 13.530 | 0.643 | 0.207 | 3.107 | 1.047 | 0.509 | 2.056 |
| **IL7R** | 71.232 | 87.841 | -16.609 | 1.845 | 2.281 | 0.809 | 1.334 | 1.075 | 1.241 |
| **IL2RG** | 79.603 | 44.816 | 34.787 | 1.514 | 0.523 | 2.896 | 0.902 | 0.634 | 1.424 |
| **IL2RA** | 5.583 | 22.323 | -16.740 | 0.077 | 0.306 | 0.253 | 0.353 | 0.633 | 0.558 |
| **CCR7** | 36.358 | 54.025 | -17.667 | 0.646 | 0.906 | 0.713 | 0.930 | 0.945 | 0.984 |
| **SELL** | 44.557 | 51.701 | -7.144 | 0.816 | 0.776 | 1.052 | 0.993 | 0.838 | 1.185 |
| **LEF1** | 42.421 | 77.520 | -35.099 | 0.694 | 1.961 | 0.354 | 0.884 | 1.249 | 0.707 |
| **TCF7** | 58.726 | 82.716 | -23.991 | 1.061 | 1.768 | 0.600 | 0.988 | 1.003 | 0.984 |
| **CXCR3** | 23.658 | 2.543 | 21.115 | 0.343 | 0.023 | 14.711 | 0.654 | 0.153 | 4.271 |
| **TBX21** | 20.053 | 16.454 | 3.599 | 0.293 | 0.188 | 1.560 | 0.617 | 0.458 | 1.348 |
| **CCR4** | 6.300 | 10.997 | -4.698 | 0.083 | 0.118 | 0.701 | 0.345 | 0.362 | 0.953 |
| **GATA3** | 30.796 | 34.116 | -3.320 | 0.413 | 0.407 | 1.016 | 0.660 | 0.621 | 1.062 |
| **RORC** | 3.010 | 5.315 | -2.305 | 0.035 | 0.058 | 0.613 | 0.211 | 0.263 | 0.803 |
| **CXCR5** | 2.336 | 3.799 | -1.463 | 0.029 | 0.038 | 0.761 | 0.195 | 0.203 | 0.962 |
| **FOXP3** | 4.150 | 4.576 | -0.427 | 0.069 | 0.054 | 1.291 | 0.357 | 0.266 | 1.343 |
| **GZMA** | 42.199 | 13.259 | 28.940 | 0.977 | 0.143 | 6.834 | 1.216 | 0.393 | 3.092 |
| **GZMB** | 23.930 | 6.860 | 17.071 | 0.564 | 0.071 | 7.995 | 1.064 | 0.279 | 3.814 |
| **GZMH** | 30.381 | 9.857 | 20.523 | 0.795 | 0.109 | 7.318 | 1.257 | 0.352 | 3.572 |
| **GZMK** | 24.009 | 8.501 | 15.508 | 0.567 | 0.092 | 6.172 | 1.069 | 0.324 | 3.301 |
| **GZMM** | 54.619 | 5.594 | 49.025 | 0.922 | 0.053 | 17.364 | 0.938 | 0.232 | 4.044 |
| **PRF1** | 41.324 | 22.004 | 19.320 | 0.831 | 0.271 | 3.064 | 1.090 | 0.551 | 1.978 |
| **GNLY** | 19.422 | 18.866 | 0.557 | 0.713 | 0.317 | 2.249 | 1.518 | 0.715 | 2.124 |
| **TRAV1-2** | 3.311 | 0.410 | 2.901 | 0.068 | 0.004 | 17.078 | 0.390 | 0.065 | 6.002 |
| **SLC4A10** | 0.853 | 2.932 | -2.079 | 0.011 | 0.045 | 0.255 | 0.130 | 0.285 | 0.456 |
| **CX3CR1** | 20.232 | 17.309 | 2.923 | 0.333 | 0.226 | 1.471 | 0.698 | 0.537 | 1.300 |
| **ITGAE** | 10.614 | 7.336 | 3.278 | 0.130 | 0.074 | 1.748 | 0.396 | 0.284 | 1.393 |
| **PDCD1** | 4.866 | 3.815 | 1.051 | 0.061 | 0.036 | 1.693 | 0.285 | 0.194 | 1.465 |
| **LAG3** | 11.245 | 1.917 | 9.328 | 0.154 | 0.018 | 8.414 | 0.457 | 0.140 | 3.274 |
| **B3GAT1** | 0.681 | 6.073 | -5.393 | 0.009 | 0.069 | 0.123 | 0.104 | 0.293 | 0.355 |

**Supplementary Table 3: Overview of transcripts analysed in figure 4.**

Percentage of cells with expression of transcripts for 10X and Parse, and the ratio between the two. Mean expression of transcripts for 10X and Parse, and the ratio between the two. Standard deviation of transcripts for 10X and Parse, and the ratio between the two.
